# Inflammatory Cytokines Are Associated with Cognitive Dysfunction and Depressive State during Acute Bacterial Infections and the Recovery Phase

**DOI:** 10.3390/ijms241814221

**Published:** 2023-09-18

**Authors:** Mónica Arias-Colinas, Alfredo Gea, Ahmed Khattab, Michael Vassallo, Stephen C. Allen, Joseph Kwan

**Affiliations:** 1 Department of Preventive Medicine and Public Health, University of Navarra, 31008 Pamplona, Spain; maricol@unav.es; 2 IdiSNA, Navarra Institute for Health Research, 31008 Pamplona, Spain; 3 Biomedical Research Network Center for Pathophysiology of Obesity and Nutrition, (CIBEROBN), Carlos III Health Institute, 28029 Madrid, Spain; 4 Faculty of Health and Social Sciences, Bournemouth University, Bournemouth BH8 8GP, UK; akhattab@bournemouth.ac.uk (A.K.); michael.vassallo@uhd.nhs.uk (M.V.); drscallen@aol.com (S.C.A.); 5 Department of Medicine for Older People, Royal Bournemouth Hospital, Bournemouth BH7 7DW, UK; 6 Department of Brain Sciences, Imperial College, London W12 0NN, UK

**Keywords:** cytokines, sickness behavior, acute bacterial infections

## Abstract

During a bacterial infection, individuals may present with behavioral changes referred to as sickness behavior, which has been suggested is induced by the inflammatory markers that are released because of the infective immunological challenge. However, few studies have explored this multidimensional phenomenon in naturally occurring conditions. A longitudinal observational study was conducted to explore the role of inflammatory cytokines in mediating the sickness behavior during a bacterial infection. There were 13, 11 and 37 participants in the infection, hospital control and healthy groups, respectively. They were all followed up for 6 weeks and their inflammatory markers were quantified throughout those weeks. Cognitive function and depressive state were assessed by means of the Mini-Mental State Examination (MMSE) and Cornell Scale for Depression in Dementia (CSDD). Reductions in proinflammatory markers C-Reactive protein (CRP), interleukin – 6 (IL6) and tumor necrosis factor-α (TNFα) and increments in anti-inflammatory markers (interleukin – 4 (IL4)) were associated with an improvement in CSDD and MSEE in patients recovering from a bacterial infection. The correlation between inflammatory makers and CSDD was statistically significant for the CRP (r = 0.535, *p* = 0.001), the IL6 (r = 0.499, *p* < 0.001), the TNFα (r = 0.235, *p* = 0.007) and the IL4 (r = −0.321, *p* = 0.018). Inflammatory cytokines may mediate sickness behavior during acute illness. These results may enhance the understanding of the pathophysiology and potential treatment strategies to palliate this sickness behavior.

## 1. Introduction

During an acute bacterial infection, individuals may present with a number of behavioral changes, which Hart in 1988 [1] referred to as sickness response or sickness behavior. Those changes have been studied by numerous authors. Miller et al. [2], Morris et al. [3], and Tracey [4] state that this sickness behavior is a set of symptoms that include lassitude, anorexia, social withdrawal, joints and muscle aches, malaise, changes in cognition, depression and isolation. It was originally thought that this response was just a component of the uncomfortable process of the acute infection itself and consequently it was usually ignored by health professionals [5,6]. However, Dantzer et al. [7] and McCusker and Kelle [8] state that it is now known that the sickness behavior is part of a complex motivational mechanism by which the body reorganizes its priorities to combat the infection by making the environment inhospitable to pathogens. Anders et al. [9] further explain that this sickness behavior is believed to be an adaptive process during infections by which the increase in body temperature and the aforementioned symptoms lead to a saving energy state which slows down the infecting organisms’ replicating process and enhances the response of the immune system.

Pavlov and Tracey [10] suggest this sickness response is thought to be mediated by the inflammatory cytokines released during acute illness through a fast neural pathway and a humoral route. When cytokines reach a high enough level, they are released into the bloodstream, and they can inform the central nervous system (CNS) that an inflammation has occurred in the periphery [11]. This is possible because blood-borne cytokines such as TNFα and IL-1 can cross the blood-brain barrier at the circumventricular organs and the choroid plexus where the blood-brain barrier is more permeable and enter the cerebrospinal fluid to interact thereafter with CNS tissue [12]. This humoral route appears to be the most common immune-to-brain communication path when cytokine levels are high enough and when there is a systemic immune challenge [11]. However, even when cytokine levels are not high enough to be released into the bloodstream, information regarding the acute process in the periphery can be conveyed to the CNS via the afferent vagus nerve (neural pathway) [13]. In the event of, for example, an infection, immune innate cells such as macrophages and monocytes secrete IL1, which can thereafter stimulate the production of other proinflammatory cytokines (IL6 and TNFα) and anti-inflammatory cytokines such as IL4 and IL10. According to Kanashiro et al. [14] and Park et al. [15] these peripherally released cytokines activate the afferent pathway of the vagus nerve, which will convey to the CNS that an infection has happened in the periphery. Furthermore, peripheral cytokine action on the CNS appear to increase the production of centrally secreted cytokines [15]. These centrally produced cytokines have been suggested to be involved in the autonomic, endocrine, metabolic and sickness response to infection [8,16,17].

There are numerous experimental studies that explore the association between proinflammatory cytokines and sickness behavior [13]. Most of these studies attempt to understand the immunological basis of the sickness response by inducing an inflammatory response via the administration of an endotoxin lipopolysaccharide (LPS) and quantifying the levels of cytokines and evaluating the behavioral response after the peripheral immune challenge [16,18]. Vollmer-Conna et al. [19] already identified that advances had been made through experimental studies that revealed that the increased levels of cytokines as a result of an immune challenge may lead to sickness behavior. However, these authors identified a need for further evaluation of the sickness behavior phenomenon and its correlation to inflammatory markers in a naturally occurring inflammatory state. They therefore conducted a study to explore these correlations in a human cohort by evaluating the symptoms of sickness behavior and the levels of inflammatory markers at the enrolment of the study and they concluded that pro-inflammatory cytokines IL-1β and IL6 were indeed associated with the sickness response. It is, however, worth highlighting that Vollmer-Conna et al. [19] focused on three specific infections (Ross River virus, Epstein–Barr virus and Q fever) and therefore it would be interesting to find out if those correlations exist in other more prevalent and commonly found infections. Furthermore, they had one data collection point, which did not allow for the evaluation of the sickness response and the inflammatory markers over time as the participants were recovering from the infectious process. To the best of our knowledge, there is a need for prospective or longitudinal studies on the correlation between inflammatory cytokines and cognitive dysfunction and sickness behavior that a person may present when recovering from an acute bacterial infection.

The present study was carried out to determine the role of inflammatory cytokines in mediating cognitive dysfunction and a depressive state in the context of sickness behavior during the recovery phase of an acute bacterial infection.

## 2. Results

### 2.1. Characteristics of Participants

Sixty-one participants met the inclusion criteria and were enrolled in this study. There were 37 participants in the healthy group, 13 in the infection group, and 11 in the hospital control group. Baseline characteristics of participants are described in Table 1.

Most participants in the infection group were admitted to the hospital with pneumonia (38.5%). One individual suffered from a urinary tract infection and another one with cellulites. In addition to this, 46.15% presented with different types of infection including lower respiratory tract infection, infective exacerbation of chronic obstructive pulmonary disease, infective exacerbation of asthma and pyelonephritis. All of them were discharged from the hospital before week 6 of follow-up.

Participants in the hospital control group had been admitted to the hospital for reasons other than infection. They all presented with different conditions such as pancreatitis, gastrointestinal bleeding, irritable bowel syndrome, headaches, multiple sclerosis exacerbation and abdominal pain.

### 2.2. CSDD and MMSE

A summary of CSDD and MMSE values and proportion of participants with abnormal values are presented in Supplementary Materials Tables S1 and S2. All individuals from the healthy and hospital control group presented with a normal MMSE (a score higher than 24 throughout the study) and the vast majority of them had a normal CSDD in all weeks. On the other hand, although all participants in the infection group had a normal MMSE at all data collection points, 50% of them (*n* = 6) had an abnormal CSDD (Mean = 10.33, SD = 7.09) in week 1. This improved in week 2 as only two individuals had an abnormal value of 12 or above (Mean = 6.92; SD = 5) and in week 6, when all the participants in this group except one had a normal CSDD score (Mean = 4.80, SD = 4.47) (Tables S1 and S2). Both the CSDD and the MMSE were significantly different in week 0 between the groups (*p* = 0.0001 and *p* = 0.036, respectively). Moreover, as it can be seen in Table 2, both the CSDD and the MMSE changes between groups were also statistically significantly different (*p* = 0.001 and *p* = 0.033, respectively) and the CSDD score changed significantly in the infection group from week 1 to 6 (*p* = 0.008) (Table 2). No statistically significant difference in MMSE and the CSDD scores between the groups in week 6 was found, suggesting that all these scores went back to normal after recovery.

### 2.3. Inflammatory Markers

**Figure 1:**
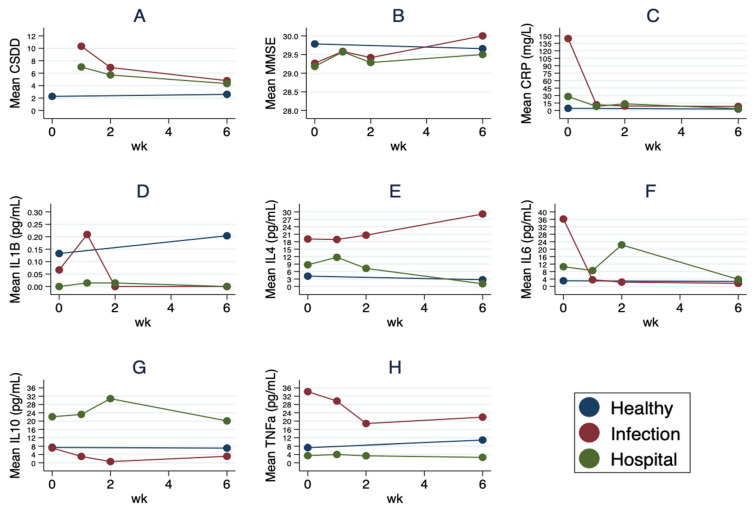
Inflammatory markers, depressive state and cognitive function in the three groups throughout the six-week period. (**A**) shows the depression state of all individuals throughout the six-week period as assessed by the CSDD and (**B**) shows the cognitive function of all individuals throughout the six-week period as evaluated by the MSEE. (**C**) displays the progression of the CRP (mg/L) in all participants throughout the six-week period, (**D**,**F**,**H**) the trends of proinflammatory cytokines (pg/mL) and (**E**,**G**) the trends of anti-inflammatory cytokines in all participants throughout the six-week period.

A summary of blood cytokines values and proportion of participants with abnormal values are presented in Supplementary Tables S3 and S4. The pro-inflammatory markers profile (CRP, IL1, IL6 and TNFα) was generally abnormal in the infection group in week 0 and those values seemed to improve throughout the weeks. Similarly, CRP and IL6 values in the hospital control group were also abnormal at the first data collection point and they improved slightly by week 6. As expected, the inflammatory markers profile was generally normal in the healthy control group (Figure 1 and Tables S3 and S4). As it can be observed in Table 3, a statistically significant difference was found in the CRP, IL6, IL10 and TNFα levels between the groups during the first visit (week 0) (*p* = 0.0001, *p* = 0.0004, *p* = 0.009 and *p* = 0.0003 respectively), with the infection group reporting generally the highest levels (CRP, Mean = 144.7, SD = 107.2; IL6, Mean = 36.2; SD = 57.2; IL10, Mean = 7.16, SD = 15.47; and TNFα, Mean = 34.21, SD = 39.73) (Table S3). The levels of IL1 showed a statistically significant difference between groups in week 6 (*p* = 0.051) and so did the levels of IL10 (*p* = 0.0006) and TNFα (*p* = 0.029). In addition to this, the change of CRP (*p* = 0.0001), IL4 (*p* = 0.009), IL6 (*p* = 0.001) and TNFα (*p* = 0.002) between groups was also significantly different (Table 3). When the change over the weeks was examined, the results showed that the CRP changed significantly in the infection group (*p* = 0.004) and nearly significantly in the hospital group (*p* = 0.063) and so did the IL4 in the healthy group (*p* = 0.007) and in the hospital group (*p* = 0.031). Furthermore, the levels of IL6 changed significantly over the 6 week period in the infection group (*p* = 0.004) and the levels of TNFα also showed significant changes over the weeks in the healthy group (*p* = 0.013) and in the infection group (*p* = 0.0098) (Table 3).

### 2.4. The Correlation between Inflammatory Markers and Sickness Behavior

**Figure 2:**
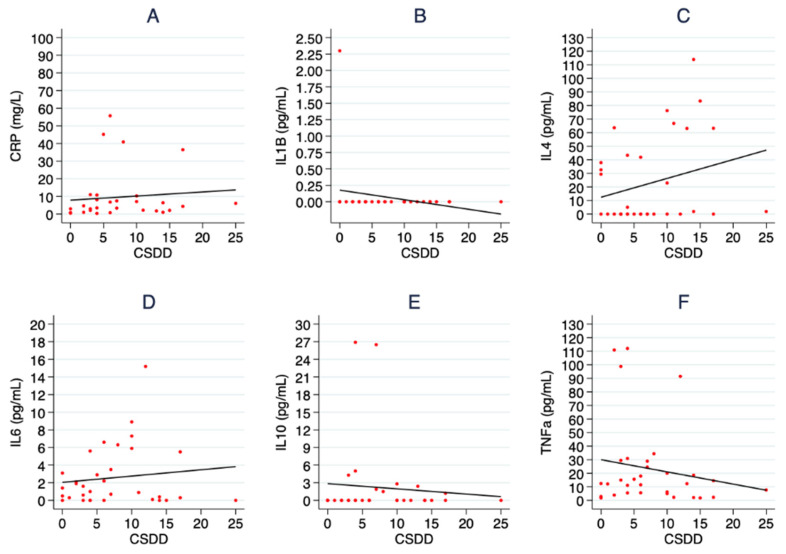
Correlations between inflammatory markers and depressive state in the infection group. In (**A**) the association between CRP and the CSDD is shown, in (**B**,**D**,**F**) the same correlation is displayed for the proinflammatory cytokines (IL1, IL6 and TNFα) and in (**C**,**E**) the association between anti-inflammatory cytokines (Il4 and Il10) and the CSDD for the participants in the infection group is shown.

The within-subject correlations (r) between the levels of inflammatory markers and sickness behavior were explored in all participants (Table 4) and it was observed that all proinflammatory markers, except IL1, presented with a positive correlation with the CSDD (CRP, r = 0.317, 0.118 to 0.484, *p* < 0.001; IL6, r = 0.336, 0.179 to 0.494, *p* < 0.001 and TNFα, r = 0.235, 0.058 to 0.411, *p* = 0.007). The same positive correlations or even stronger were found when the correlations were explored in the infection group, but the statistical significance was lost for the TNFα (CRP, r = 0.535, 0.181 to 0.771, *p* = 0.001; IL6, r = 0.499, 0.257 to 0.707, *p* < 0.001 and TNFα, r = 0.244, −0.101 to 0.585, *p* = 0.119) (Figure 2 and Table 4). IL1, however, did not show any statistically significant correlations to either the CSDD in all participants (r = −0.004, −0.148 to 0.182, *p* = 0.956) or in the infection group (r = −0.017, −0.152 to 0.089, *p* = 0.766). Furthermore, IL6 presented with a statistically significant correlation with the MMSE in all participants (r = −0.172, −0.314 to 0.020, *p* = 0.042) and IL4 showed a negative correlation with the CSDD in the infection group (r = −0.321, −0.571 to −0.043, *p* = 0.018) which was not statistically significant in all participants (r = 0.016, −0.150 to 0.176, *p* = 0.850) (Table 4).

## 3. Discussion

In our longitudinal study, reductions in pro-inflammatory markers (CRP, IL6 and TNFα) and increments in anti-inflammatory markers (IL4) were associated with an improvement in CSDD and MMSE levels in patients recovering from a bacterial infection (Figure 2 and Table 4).

The results of this study show that the levels of proinflammatory markers generally decreased throughout the weeks together with the CSDD scores (which therefore became more normal) (Tables S1 and S3). When this was explored further, the results showed that there was a moderate positive association between all proinflammatory markers, except IL1, and the CSDD in all participants and these associations were statistically significant. Interestingly, these associations were also found when the correlations were explored in the infection group (Table 4). This concurs with what was found by Vollmer-Conna et al. [19], who also found out that there was a positive association between sickness response symptoms and the levels of IL1β and Il6.

Vollmer-Conna et al. [19] explored only such associations at one point in time and therefore did not provide an insight into the progression over time as the individual recovered from the infectious process. The present study adds some insight into this progression as the results show a positive association of changes in cytokines and changes in the CSDD values over the six-week period. This positive correlation between pro-inflammatory markers and the CSDD score would have been expected as the CSDD scores should be improving as individuals recovered from the infection. Individuals were admitted with an acute bacterial infection and therefore, they presented with a higher percentage of abnormal levels of proinflammatory markers on admission. Therefore, and according to Shattuck and Muehlenbein [11], those cytokines would have conveyed to the CNS that an infection had happened in the periphery. This would lead to the sickness response [20]. However, as individuals were being treated for the bacterial infection, the levels of proinflammatory markers decreased to the point that most individuals presented with normal levels in the last visit (week 6). This therefore would decrease the sickness response, which would be manifested by a lower CSDD in the last visit (week 6). Additionally, a negative correlation between the pro-inflammatory markers and the MMSE was found, and this would have been expected for the same reasons. Individuals should be improving their MMSE score as the infection process was being managed. This negative association between proinflammatory markers and MMSE scores was in fact found in all participants although only statistically significant in the case of the IL6. It may have been that the sample size was not large enough for those correlations to be fully unveiled.

IL4 was negatively associated with the CSDD in the infection group (Table 4), which could mean that as IL4 values increased throughout the weeks, the CSDD score decreased (and therefore became more normal or improved). It is worth highlighting that when the levels of IL4 were analyzed in the infection group in week 0, 26.67% of the individuals presented with abnormal values and that worsened throughout the weeks as 40% of the participants had abnormal IL4 values in week 6. It could therefore be questioned why IL4 levels did not improve after 6 weeks. IL4 belongs to the anti-inflammatory cytokines group and according to Song et al. [21] their release post-inflammation is fundamental in order to downgrade the effect of the proinflammatory cytokines. This is further supported by Anovazzi et al. [22] and Lubis et al. [23] who explain that the release of anti-inflammatory cytokines such as IL4 is part of the body’s homeostatic mechanism to regulate the magnitude of the local inflammatory response. Shattuck and Muehlenbein [11] explain that the release of IL4 post-inflammation will suppress IL1 and TNFα secretion by macrophages, therefore regulating the magnitude of the inflammatory response. Therefore, a higher level of IL4 would be expected during the early stages of an infection as IL4 would be released to regulate the inflammatory process. However, once the infection is treated and the accompanying inflammatory process reduced, the levels of IL4 should decrease [24]. The fact that on this occasion IL4 levels did not go back to normal levels is not well understood and may require further exploration in the future, as it may explain the reason why there was a negative association between IL4 and the CSDD in the infection group. For this study, individuals were scrutinized for other conditions as part of the admission criteria and at the time of the follow-up appointments no individuals were suffering from any other condition that had not been previously identified. One may therefore wonder whether participants failed to mention an underlying inflammatory process or condition as they did not deem it necessary to disclose that information. For example, elevated levels of IL4 are commonly found in patients with atopic dermatitis [25,26] and allergic rhinitis [27]. It is therefore plausible to suggest that individuals in this group may have been suffering from a chronic inflammatory process of this sort, which they failed to mention as they did not consider it necessary. This, according to Woodward et al. [28], could be a potential explanation for the relatively high levels of IL4 throughout the 6 weeks as IL4 is released post-inflammation to down-regulate the inflammatory response.

Consequently, in order to fully understand the correlation between inflammatory markers and the sickness response in an acute bacterial infection, a larger-scale study could be carried out with a larger sample size where potential participants are further scrutinized for chronic inflammatory conditions that could potentially alter the profile of those markers.

A limitation of the present study was therefore the reduced sample size. Recruiting the infection group and the hospital control group proved to be a difficult task mainly due to the exclusion criteria for the study, as only a very small number of individuals were eligible to participate and were approached by the researcher to be invited and informed about the study. Moreover, as individuals were being approached at a very early stage of the admission process, although they were hemodynamically stable, they were still unwell and therefore the great majority did not want to participate at that stage. In addition to this, some of those who were considering participating in the study declined to do so once they knew they had to meet the researcher for three follow-up appointments. Consequently, the author acknowledges that the smaller sample size may have affected the interpretation and the validity of the obtained results. It is therefore for this reason that in the future, a larger-scale study can be carried out to further explore these issues in greater detail.

Another limitation of the present study is that sickness behavior is a multidimensional phenomenon, and it is therefore a challenge to assess all its elements. No clinical scale to date has been thoroughly validated for its evaluation. Only one scale, developed by Andreasson et al. [29], was identified. These authors recognized the lack of a psychometric assessment tool for the sickness behavior in response to an infection. They consequently developed a 10 item questionnaire that covered the most relevant aspects of such a response. The questionnaire appeared to be valid and reliable in identifying and assessing the most relevant aspects of the sickness behavior. However, considering the novelty of the assessment instrument, Andreasson et al. [29] recommended further research was needed to test the validity and reliability of their questionnaire. It is for this reason that for the present study it was decided to focus on the evaluation of the two aspects of this sickness behavior, the cognitive state and the signs and symptoms of a depressed mood, on the basis that those aspects cover most of the nonspecific symptoms described in the literature as typical of sickness behavior.

## 4. Materials and Methods

This was a longitudinal observational study to explore the association between inflammatory biomarkers and the cognitive function and depressive state in the context of the sickness behavior during an acute bacterial infection. The study was conducted at the X Hospital in the X country and it was approved by the Local Research Ethics Committee. All participants received a verbal explanation about the study and a participant information sheet. Having read and understood all the information given, participants signed a consent form to participate in the study. Participants were reassured that anonymity and confidentiality would be kept at all times and that their participation in the study would not interfere with their medical treatment. The study was conducted according to the principles stated in the Declaration of Helsinki.

### 4.1. Inclusion and Exclusion Criteria

The general inclusion criteria were: (1) any person who was over 18 years old and was able to freely agree to participate; and (2) participants needed to be clinically stable by the time the first blood test and cognitive assessment were completed (24 h from hospital admission). The criteria of stability were the patient being adequately hydrated with improving biochemical profile, apyrexial (Ta < 37.8 °C), systolic blood pressure > 90 mmHg, heart rate ≤ 100 beats/minute, respiratory rate ≤ 20 respirations/minute and pulse oximetry ≥ 90%, treatment started and well enough to participate in the assessment protocol [30,31,32,33].

The exclusion criteria were: (1) patients with significant communication difficulties (e.g., severe aphasia); (2) patients with severe cognitive impairment (mini-mental state examination (MMSE) 24 or less); (3) patients with depression.

### 4.2. Subjects and Groups

Those subjects who met the inclusion criteria and agreed to participate were included in one of three different groups:

Acute bacterial infection: 13 patients were recruited who had been admitted to the hospital with a diagnosis of an active and symptomatic bacterial infection. Participants in this group had signs and symptoms of the infection and required inpatient treatment. All those patients who were seriously ill or were unlikely to recover sufficiently to take part in the study within the subsequent 6 weeks were not approached. At the time of their first blood test and cognitive assessment (24 h from hospital admission), participants were clinically stable (i.e., adequately hydrated with improving biochemical profile, apyrexial, treatment started, and well enough to participate in the assessment protocol).

Hospital control group: 11 participants admitted with a definite diagnosis other than a bacterial infection were included in this study. The patients in this group were selected as closely as possible for age and gender (similar to the infection group).

Healthy control group: 37 subjects were included in this group. Individuals in this group had not had a bacterial infection requiring treatment for the previous 6 months. Participants in this group were selected with age and sex as similar as possible to the infection group.

The sample size was calculated considering the primary objective of the study. Twelve participants in each group would allow identifying differences of 60 mg/L and the C-Reactive Protein (CRP) with a statistical power of 90%. These differences are lower than expected even when the two groups of hospitalized participants are compared. The same number of participants per group would show 80% statistical power when the TNFα is compared in the hospitalized groups, with differences of 30 pg/mL.

### 4.3. Inflammatory Markers, Cognitive Function and Depressive State

Serum blood cytokines (IL-1, IL4, IL-6, IL10 and TNFα) and CRP were measured at 24 h from hospital admission (week 0) and thereafter at the follow-up visits (week 1, week 2 and week 6) (in the healthy group CRP and blood inflammatory cytokines were measured in week 0 and 6 weeks later). Cytokines were measured by using the traditional enzyme-linked immunosorbent assay (ELISA). According to Chiswick et al. [34] and Leng et al. [35], the ELISA immunoassay is based on a well that is coated with an antibody. The antigen (cytokine) that needs to be measured is then added onto it and a detection antibody linked to an enzyme is then incubated with the antigen-antibody in the well. The substrate agent is then added and that will create fluorescent or luminescent products [36]. The concentration of the antigen was previously determined by a set of pre-run clinical calibrations that indicated the concentration of the antigen (cytokine) in relation to the fluorescent intensity [36]. Leng et al. [35] concur that the ELISA kits have a very high specificity and sensitivity, and the results are highly reproducible, which makes this kit one of the most popular ones in clinical laboratories. 

Sickness behavior is a multidimensional phenomenon, and it is therefore a challenge to assess all its elements. It is therefore for this reason that for the present study it was decided to focus on the evaluation of two aspects of this sickness behavior, the cognitive state and the signs and symptoms of a depressed mood, on the basis that those aspects cover most of the nonspecific symptoms typical of sickness behavior. In order to evaluate these two aspects, two well-recognized clinical scales were used: the MMSE and the Cornell Scale for depression in dementia (CSDD).

The MMSE is a popular, brief and easy-to-use scale to assess the cognitive function of an individual by means of nineteen items that provide information on orientation, registration, attention and calculation, recall and language and praxis [34,35]. The CSDD is an assessment tool to identify signs and symptoms of depression [3,36,37].

The cognitive state and the depressive signs and symptoms in the context of sickness behavior were therefore assessed for the three groups using the MMSE (week 0, 1, 2 and 6) and the CSDD (weeks 1, 2 and 6). A MMSE was considered abnormal if the score was 24 or below [24] and the CSDD was considered abnormal if the score was 12 or above [38,39,40].

### 4.4. Data Analysis

Analyses were performed using STATA/SE version 15.0 (StataCorp). We used two-sided *p*-values, and the statistical significance threshold was set a priori at 0.05.

Baseline characteristics of participants were described using mean and standard deviation for quantitative variables and absolute frequency and percentages for qualitative variables. Differences between-groups in MMSE, CSDD and blood cytokines at each time-point were assessed using the Kruskal–Wallis test. Changes between baseline and week 6 were assessed with the Wilcoxon test. To study the correlation between MMSE, CSDD and blood cytokines, repeated measures correlation were performed. The within-subject correlation coefficients are presented to account for the correlation between changes in both assessed variables. The 95% confidence intervals (CI) were the 2.5th and 97.5th percentiles of the distribution obtained from a nonparametric bootstrap with 200 samples.

## 5. Conclusions

This study found an association between inflammatory cytokines and cognitive dysfunction and sickness behavior during acute infection as well as during the recovery phase from such an infection. This suggests that inflammatory cytokines may mediate in these processes. Further mechanistic studies are needed to confirm this hypothesis. These results may enhance the understanding of the pathophysiology and potential treatment strategies to palliate this sickness behavior in response to an infective immunological challenge.

## Figures and Tables

**Table 1:** Details of patient groups.

	Healthy Group	Infection Group	Hospital Control Group
Number	37	13	11
Mean age (years) Range	52.4 (33–76)	47.9 (24–69)	54.5 (20–84)
Females	67.6% (25/37)	46.2% (6/13)	63.6% (7/11)
Males	32.4% (12/37)	53.8% (7/13)	36.4% (4/11)
Smokers	16.2% (6/37)	38.5% (5/13)	36.4% (4/11)
Alcohol > 14 Units/week	5.4% (2/37)	7.7% (1/13)	18.2% (2/11)
Independent with ADLs	100% (37/37)	100% (13/13)	100% (11/11)
Mean white blood cells × 10^9^/L (SD) *	-	15.4 (5.9)	9.1 (3)
Mean CRP mg/L (SD) *	-	145 (107)	-
Mean oxygen sat % (SD) *	-	96 (3)	92.3%
Pyrexia °C (%) *	-	61.5% (8/13)	0%

* Parameters on hospital admission.

**Table 2:** Kruskal–Wallis (between-groups *p*-value) for the differences between CSDD and MMSE at each time-point and Wilcoxon (Within-group *p*-value) for the assessment of the changes in CSDD and MMSE between baseline and week 6.

		Week 0/1	Week 6	Change	Within-Group *p*-Value
CSDD	Healthy group	2.27 (2.38)/37	2.60 (2.64)/35	+0.23 (2.13)/35	*p* = 0.54
	Hospital group	7.00 (4.28)/7	4.33 (2.88)/6	−1.29 (1.83)/6	*p* = 0.063
	Infection group	10.33 (7.09)/12	4.80 (4.47)/10	−4.20 (6.16)/10	*p* = 0.008
	Between-groups *p*-value	*p* = 0.0001	*p* = 0.166	*p* = 0.001	
		Week 0	Week 6	Change	Within-group *p*-value
MMSE	Healthy group	29.8 (0.48)/37	29.7 (0.54)/35	−0.11 (0.68)/35	*p* = 0.33
	Hospital group	29.2 (0.98)/11	29.5 (0.84)/6	0.16 (0.75)/6	*p* > 0.999
	Infection group	29.3 (1.10)/15	30.0 (0)/10	+0.7 (1.06)/10	*p* = 0.125
	Between-groups *p*-value	*p* = 0.036	*p* = 0.123	*p* = 0.033	

**Table 3:** Kruskal–Wallis (between-groups *p*-value) for the differences between inflammatory markers’ levels at each time-point and Wilcoxon (Within-group *p*-value) for the assessment of the changes in inflammatory markers´ levels between baseline and week 6.

		Week 0	Week 6	Change	Within-Group *p*-Value
CRP	Healthy group	4.41 (12.1)/38	2.88 (6.1)/36	−1.65 (13.7)/36	*p* = 0.27
	Hospital group	28.1 (40.4)/11	3.8 (6.1)/6	−16.6 (32.3)/6	*p* = 0.063
	Infection group	144.7 (107.2)/13	8.2 (13.4)/10	−110.2 (100.1)/9	*p* = 0.004
	Between-groups *p*-value	*p* = 0.0001	*p* = 0.145	*p* = 0.0001	
		Week 0	Week 6	Change	Within-group *p*-value
IL1	Healthy group	0.13 (0.27)/38	0.20 (0.41)/36	+0.07 (0.47)/36	*p* = 0.43
	Hospital group	0 (0)/9	0 (0)/6	0 (0)/6	*p* = 1
	Infection group	0.07 (0.16)/15	0 (0)/10	−0.08 (0.19)/10	*p* = 0.50
	Between-groups *p*-value	*p* = 0.129	*p* = 0.051	*p* = 0.46	
		Week 0	Week 6	Change	Within-group *p*-value
IL4	Healthy group	4.16 (3.39)/38	2.73 (2.42)/36	−1.65 (3.25)/36	*p* = 0.007
	Hospital group	8.73 (12.11)/9	1.10 (0.81)/6	−5.72 (7.47)/6	*p* = 0.031
	Infection group	19.1 (28.0)/15	29.1 (41.4)/10	+12.32 (32.8)/10	*p* = 0.25
	Between-groups *p*-value	*p* = 0.54	*p* = 0.60	*p* = 0.009	
		Week 0	Week 6	Change	Within-group *p*-value
IL6	Healthy group	3.07 (3.80)/38	2.71 (1.91)/36	−0.41 (3.49)/36	*p* = 0.42
	Hospital group	10.6 (25.3)/9	3.83 (2.56)/6	+0.85 (2.42)/6	*p* = 0.44
	Infection group	36.2 (57.2)/15	1.68 (1.99)/10	−31.8 (68.4)/10	*p* = 0.004
	Between-groups *p*-value	*p* = 0.0004	*p* = 0.10	*p* = 0.001	
		Week 0	Week 6	Change	Within-group *p*-value
IL10	Healthy group	7.43 (5.51)/38	7.08 (5.79)/36	−0.34 (4.87)/36	*p* = 0.74
	Hospital group	22.10 (60.02)/9	20.12 (48.45)/6	−12.4 (25.1)/6	*p* = 0.50
	Infection group	7.15 (15.47)/15	3.15 (8.35)/10	−6.89 (21.0)/10	*p* = 0.41
	Between-groups *p*-value	*p* = 0.009	*p* = 0.0006	*p* = 0.44	
		Week 0	Week 6	Change	Within-group *p*-value
TNFα	Healthy group	7.29 (6.23)/38	10.88 (9.71)/36	+3.83 (9.58)/36	*p* = 0.013
	Hospital group	3.46 (3.39)/9	2.62 (2.10)/6	−0.82 (1.58)/6	*p* = 0.38
	Infection group	34.21 (39.73)/15	21.92 (32.70)/10	−11.7 (20.3)/10	*p* = 0.0098
	Between-groups *p*-value	*p* = 0.0003	*p* = 0.029	*p* = 0.002	

**Table 4:** Within-Subjects correlations (Spearman’s r values) and Confidence Interval at 95% between inflammatory markers and CSDD and MMSE in all participants and in the infection group only.

	All Participants		Only Infection Group	
	CSDD	MMSE	CSDD	MMSE
CRP	0.317 (0.118 to 0.484); *p* < 0.001	−0.084 (−0.258 to 0.082); *p* = 0.350	0.535 (0.181 to 0.771); *p* = 0.001	−0.227 (−0.506 to 0.112); *p* = 0.143
IL1	−0.004 (−0.148 to 0.182); *p* = 0.956	−0.097 (−0.245 to 0.063); *p* = 0.202	−0.017 (−0.152 to 0.089); *p* = 0.766	−0.242 (−0.532 to 0.145); *p* = 0.162
IL4	0.016 (−0.150 to 0.176); *p* = 0.850	−0.013 (−0.222 to 0.197); *p* = 0.901	−0.321 (−0.571 to −0.043); *p* = 0.018	−0.138 (−0.464 to 0.201); *p* = 0.426
IL6	0.336 (0.179 to 0.494); *p* < 0.001	−0.172 (−0.314 to 0.020); *p* = 0.042	0.499 (0.257 to 0.707); *p* < 0.001	−0.245 (−0.549 to 0.103); *p* = 0.128
IL10	0.000 (−0.169 to 0.194); *p* = 0.997	0.077 (−0.152 to 0.189); *p* = 0.928	−0.040 (−0.388 to 0.323); *p* = 0.836	0.090 (−0.180 to 0.332); *p* = 0.508
TNFα	0.235 (0.058 to 0.411); *p* = 0.007	−0.139 (−0.290 to 0.050); *p* = 0.109	0.244 (−0.101 to 0.585); *p* = 0.119	−0.084 (−0.404 to 0.217); *p* = 0.605

## Data Availability

The data presented in this study are available on request from the corresponding author. The data are not publicly available due to privacy.

## Supplementary Materials

The following supporting information can be downloaded at: https://www.mdpi.com/article/10.3390/ijms241814221/s1.
