# Supplementary material for: Inflammatory Cytokines Are Associated with Cognitive Dysfunction and Depressive State during Acute Bacterial Infections and the Recovery Phase
Source: Int J Mol Sci. 2023 Sep 18;24(18):14221. doi: 10.3390/ijms241814221 (PMC10532050; doi:10.3390/ijms241814221)
Supplement: Supplementary file 1 [file ijms-24-14221-s001.zip › ijms-2567513-supplementary.pdf]

**Table S1.** CSDD and MMSE Assessment in all the groups throughout the six-week period (Mean(SD)/N).

| | | Week 0 | Week 1 | Week 2 | Week 6 |
|---|---|---|---|---|---|
| CSDD | Healthy group | 2.27 (2.38) / 37 | | - | 2.60 (2.64) / 35 |
| | Hospital group | | 7.00 (4.28) / 7 | 5.71 (5.09) / 7 | 4.33 (2.88) / 6 |
| | Infection group | | 10.33 (7.09) / 12 | 6.92 (5.00) / 12 | 4.80 (4.47) / 10 |
| MMSE | Healthy group | 29.8 (0.48) / 37 | - | - | 29.7 (0.54) / 35 |
| | Hospital group | 29.2 (0.98) / 11 | 29.6 (0.79) / 7 | 29.3 (0.76) / 7 | 29.5 (0.84) / 6 |
| | Infection group | 29.3 (1.10) / 15 | 29.6 (0.67) / 12 | 29.4 (0.67) / 12 | 30.0 (0) / 10 |

**Table S2.** Percentage of patients in the three groups with an abnormal CSDD and MMSE throughout the six-week period.

| | | Week 0 | Week 1 | Week 2 | Week 6 |
|---|---|---|---|---|---|
| CSDD | Healthy group | 0% (0/37) | | - | 2.9% (1/35) |
| | Hospital group | | 14.3% (1/7) | 14.3% (1/7) | 0% (0/6) |
| | Infection group | | 50% (6/12) | 16.7% (2/12) | 10% (1/10) |
| MMSE | Healthy group | 0% (0/37) | - | - | 0% (0/35) |
| | Hospital group | 0% (0/11) | 0% (0/7) | 0% (0/7) | 0% (0/10) |
| | Infection group | 0% (0/15) | 0% (0/12) | 0% (0/12) | 0% (0/6) |

**Table S3.** Inflammatory markers quantification in all the groups throughout the six-week period (Mean (SD)/N).

| | | Week 0 | Week 1 | Week 2 | Week 6 |
|---|---|---|---|---|---|
| CRP | Healthy group | 4.41 (12.1) /38 | - | - | 2.88 (6.1) /36 |
| | Hospital group | 28.1 (40.4) /11 | 8.3 (16.2) / 5 | 13.4 (19.1) / 7 | 3.8 (6.1) / 6 |
| | Infection group | 144.7 (107.2) / 13 | 11.6 (14.6) / 10 | 16.8 (9.0) / 10 | 8.2 (13.4) / 10 |
| IL1 | Healthy group | 0.13 (0.27) / 38 | - | - | 0.20 (0.41) / 36 |
| | Hospital group | 0 (0) / 9 | 0.01 (0.04) / 7 | 0.01 (0.04) / 7 | 0 (0) / 6 |
| | Infection group | 0.07 (0.16) / 15 | 0.21 (0.69) / 11 | 0 (0) / 12 | 0 (0) / 10 |
| IL4 | Healthy group | 4.16 (3.39) / 38 | - | - | 2.73 (2.42) / 36 |
| | Hospital group | 8.73 (12.11) / 9 | 11.74 (12.41) / 7 | 7.29 (8.25) / 7 | 1.10 (0.81) / 6 |
| | Infection group | 19.1 (28.0) / 15 | 18.9 (26.9) / 11 | 20.6 (29.5) / 12 | 29.1 (41.4) / 10 |
| IL6 | Healthy group | 3.07 (3.80) / 38 | - | - | 2.71 (1.91) / 36 |
| | Hospital group | 10.6 (25.3) / 9 | 8.57 (16.7) / 7 | 22.33 (51.83) / 7 | 3.83 (2.56) / 6 |
| | Infection group | 36.2 (57.2) / 15 | 3.60 (4.53) / 11 | 2.37 (3.24) / 12 | 1.68 (1.99) / 10 |
| IL10 | Healthy group | 7.43 (5.51) / 38 | - | - | 7.08 (5.79) / 36 |
| | Hospital group | 22.10 (60.02) / 9 | 23.23 (59.15) / 7 | 30.83 (79.46) / 7 | 20.12 (48.45) / 6 |
| | Infection group | 7.15 (15.47) / 15 | 3.08 (7.95) / 11 | 0.59 (1.42) / 12 | 3.15 (8.35) / 10 |
| TNFa | Healthy group | 7.29 (6.23) / 38 | - | - | 10.88 (9.71) / 36 |
| | Hospital group | 3.46 (3.39) / 9 | 4.00 (3.47) / 7 | 3.37 (2.72) / 7 | 2.62 (2.10) / 6 |
| | Infection group | 34.21 (39.73) / 15 | 29.67 (37.33) / 11 | 18.83 (26.63) / 12 | 21.92 (32.70) / 10 |

**Table S4.** Percentage of patients in the three groups with an abnormal inflammatory marker's´values throughout the six-week period.

| | | Week 0 | Week 1 | Week 2 | Week 6 |
|---|---|---|---|---|---|
| CRP | Healthy group | 5.26% (2/38) | - | - | 2.78% (1/36) |
| | Hospital group | 45.45% (5/11) | 20% (1/5) | 42.86% (3/7) | 16.67% (1/6) |
| | Infection group | 92.31% (12/13) | 30% (3/10) | 30% (3/10) | 10% (1/10) |
| IL1 | Healthy group | 0% (0/38) | - | - | 0% (0/36) |
| | Hospital group | 0% (0/9) | 0% (0/7) | 0% (0/7) | 0% (0/6) |
| | Infection group | 0% (0/15) | 0% (0/11) | 0% (0/12) | 0% (0/10) |
| IL4 | Healthy group | 0% (0/38) | - | - | 0% (0/36) |
| | Hospital group | 11.11% (1/9) | 14.29% (1/7) | 0% (0/7) | 0% (0/6) |
| | Infection group | 26.67% (4/15) | 36.36% (4/11) | 25% (3/12) | 40% (4/10) |
| IL6 | Healthy group | 26.32% (10/38) | - | - | 27.78% (10/36) |
| | Hospital group | 33.33% (3/9) | 28.57% (2/7) | 28.57% (2/7) | 33.33% (2/6) |
| | Infection group | 80% (12/15) | 36.36% (4/11) | 25% (3/12) | 20% (2/10) |
| IL10 | Healthy group | 50% (19/38) | - | - | 38.89% (14/36) |
| | Hospital group | 22.22% (2/9) | 14.29% (1/7) | 14.29% (1/7) | 16.67% (1/6) |
| | Infection group | 13.33% (2/15) | 9.09% (1/11) | 0% (0/12) | 10% (1/10) |
| TNFa | Healthy group | 13.16% (5/38) | - | - | 16.67% (6/36) |
| | Hospital group | 0% (0/9) | 0% (0/7) | 28.57% (2/7) | 0% (0/6) |
| | Infection group | 40% (6/15) | 36.36% (4/11) | 75% (9/12) | 40% (4/10) |
